# A comparison of the local immune status between the primary and metastatic tumor in colorectal cancer: a retrospective study

**DOI:** 10.1186/s12885-018-4276-y

**Published:** 2018-04-03

**Authors:** Masatsune Shibutani, Kiyoshi Maeda, Hisashi Nagahara, Tatsunari Fukuoka, Shinji Matsutani, Shinichiro Kashiwagi, Hiroaki Tanaka, Kosei Hirakawa, Masaichi Ohira

**Affiliations:** Department of Surgical Oncology, Osaka City University Graduate School of Medicine, 1–4–3 Asahi-machi Abeno–ku, Osaka City, Osaka Prefecture 545-8585 Japan

**Keywords:** Colorectal cancer, Tumor-infiltrating lymphocyte, Primary tumor, Metastatic tumor

## Abstract

**Background:**

The anticancer immune response has been reported to correlate with cancer progression. Tumor-infiltrating lymphocytes (TILs), which are one of the indicators of host immunity, affect the tumor growth, metastasis and chemoresistance. Both TILs in the primary tumor and those in the metastatic tumor have been reported to be a useful predictor of the survival and therapeutic outcome. However, the correlation between the density of TILs in the primary and metastatic tumor is unclear. The aim of this study was to elucidate the correlation between the density of TILs in the primary and metastatic tumor.

**Methods:**

A total of 24 patients with stage IV colorectal cancer who underwent concurrent resection of the primary tumor and liver metastasis were enrolled in order to assess the correlation between the density of TILs in the primary tumor and that in the metastatic tumor. Hematoxylin and eosin (HE)-stained tumor sections were used for the evaluation of TILs. The density of TILs was assessed by the measurement of the area occupied by mononuclear inflammatory cells over the total stromal area at the invasive margin. In addition, to evaluate TIL subsets and the activation/suppression status of the lymphocytes, immunohistochemistry for CD4, CD8, Forkhead boxprotein P3 (FOXP3), programmed cell death 1 (PD-1), cytotoxic T-lymphocyte-associated protein 4 (CTLA4), inducible T-cell co-stimulator (ICOS), Glucocorticoid induced tumor necrosis factor receptor related protein (GITR), Human Leukocyte Antigen - antigen D Related (HLA-DR) and Granzyme B was performed, and the number of immunoreactive lymphocytes was counted.

**Results:**

According to the evaluation using the HE-stained sections, the density of tumor-infiltrating mononuclear inflammatory cells in the primary tumor was significantly associated with that in the metastatic tumor. In addition, according to the immunohistochemistry evaluation, the density of CD4^+^, CD8^+^ and FOXP3^+^ TILs in the primary tumor and that in the metastatic tumor were significantly correlated with that in the metastatic tumor. Furthermore, the activation/suppression marker values of the lymphocytes (i.e.*,* such as PD-1, ICOS, Granzyme B and the PD-1/CD8 ratio) in the primary tumor were correlated with values in the metastatic tumor.

**Conclusions:**

The local immune status of the primary tumor was revealed to be similar to that of the metastatic tumor. This suggests that the evaluation of the local immunity of the primary tumor may be a substitute for the evaluation of the local immunity of the metastatic lesion. Therefore, information on the primary tumor may be useful when considering treatment strategies for metastatic lesions.

## Background

The immune status of the host has been recognized to be correlated with the cancer progression in patients with various types of cancer. Tumor-infiltrating lymphocytes (TILs), which are one of the indicators of host immunity, affect the tumor growth, metastasis and chemoresistance. Regarding the primary tumor, a high density of TILs was revealed to be correlated with better survival rates [1–3], and TILs were reported to be superior to TNM classification as a predictor of the survival in patients with colorectal cancer (CRC) [2]. In addition, a high density of TILs was also reported to be correlated with a better efficacy of neoadjuvant chemoradiotherapy in patients with locally advanced rectal cancer [4].

TILs in the metastatic tumor as well as those in the primary tumor were reported to be a useful predictor of the therapeutic outcome, although relatively few reports on TILs in metastatic tumors have been published compared to reports on TILs in primary tumors. A high density of TILs in the metastatic tumor was reported to be correlated with better relapse-free and overall survival rates after resection of the metastatic tumor [5, 6] and a better chemotherapeutic outcome [7]. However, the correlation between the local immune status of the primary tumor and that of the metastatic tumor has been unclear.

The aim of this study was to assess the correlation between the local immune status of the primary tumor and that of the metastatic tumor.

## Methods

### Patients

We retrospectively reviewed a database of 24 patients with stage IV CRC who underwent concurrent resection of the primary tumor and the metastatic liver tumor at the Department of Surgical Oncology of Osaka City University between 2003 and 2016. Patients who underwent preoperative therapy, such as chemotherapy and radiotherapy, were excluded from this study.

### Selection of the metastatic liver tumor for an evaluation

In case of multiple liver metastases, we selected the largest metastatic tumor for the evaluation of TILs.

### The evaluation of TILs using stained sections

**Fig. 1:**
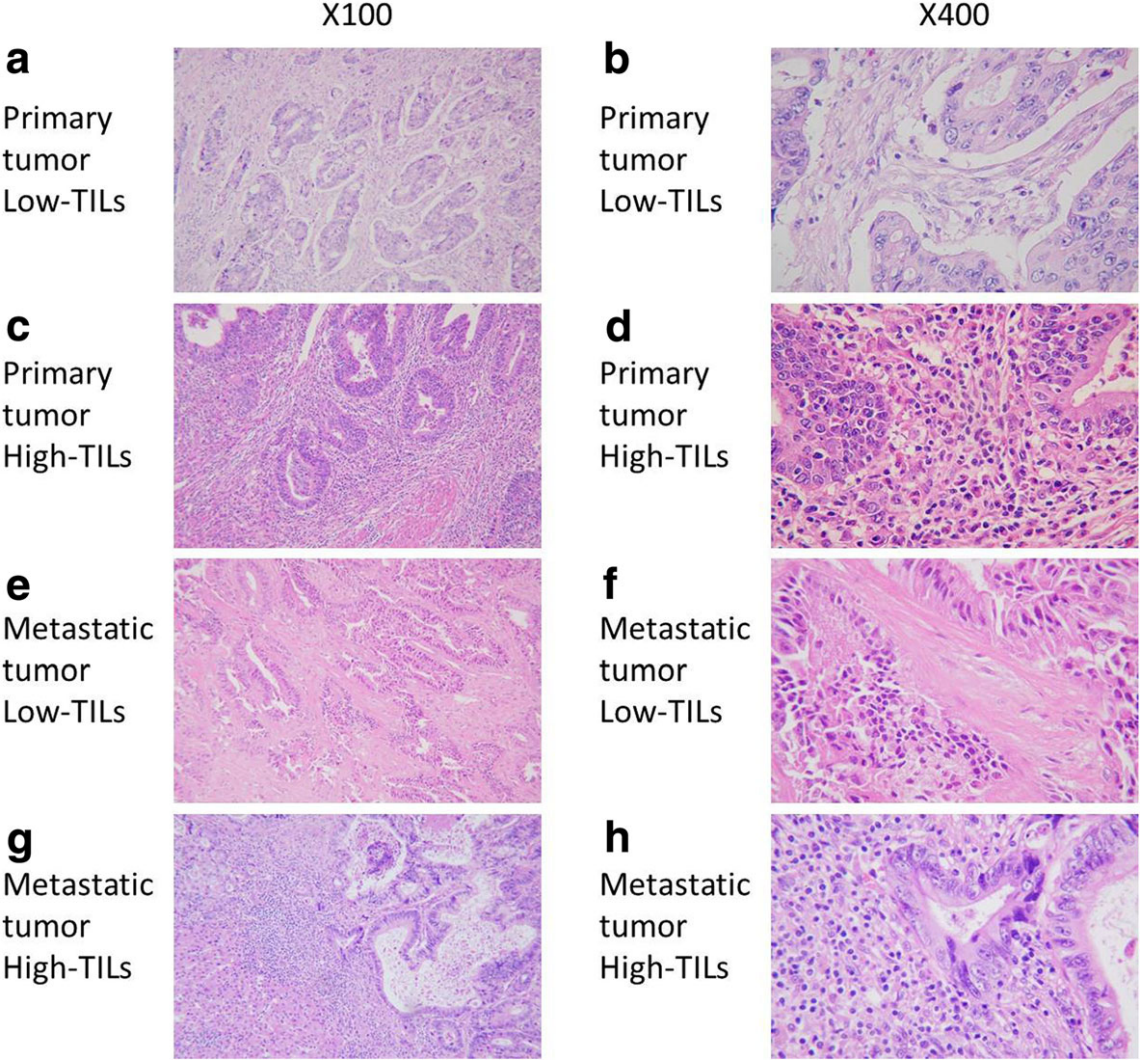
Representative pictures of intratumoral inflammatory cell infiltration by hematoxylin and eosin staining. Low density of inflammatory cell infiltration in the primary tumor (**a**: × 100, **b**: × 400). High density of inflammatory cell infiltration in the primary tumor (**c**: × 100, **d**: × 400). Low-density inflammatory cell infiltration in the metastatic tumor (**e**: × 100, **f**: × 400). High-density inflammatory cell infiltration in the metastatic tumor (**g**: × 100, **h**: × 400)

The density of TILs in hematoxylin and eosin (HE)-stained sections was evaluated according to the recommendations proposed by an International TILs Working Group [8]. In brief, the density of TILs was assessed by the measurement of the area occupied by mononuclear inflammatory cells over the total stromal area at the invasive margin (Fig. 1). Only the stromal area was evaluated, and areas occupied by cancer cells were excluded for the evaluation. A full assessment of average TILs was used, and hotspots did not receive focus. We set 50% as the cut-off value for the evaluation of TILs using the HE-stained section according to the verification study of the recommendations proposed by an International TILs Working Group [9] and classified the patients into high- and low-TIL groups based on the cut-off value.

### Immunohistochemistry

Surgically resected specimens for all enrolled patients were retrieved to perform the immunohistochemistry. Immunohistochemistry was done as previously described [10]. Primary specific antibodies for CD4 (4B12, 1:80 dilution; Dako, Glostrup, Denmark), CD8 (C8/144B, 1:100 dilution; Dako), FOXP3 (236/E7, 1:100 dilution; Abcam, Cambrige, UK), PD-1 (NAT105, 1:50 dilution; Abcam), CTLA4 (UMAB249, 1:200 dilution; OriGene Technologies, Rockville, US), ICOS (EPR20560, 1:500 dilution; Abcam), GITR (D919D, 1:400 dilution; Cell Signaling Technologies, Beverly, US), HLA-DR (L243, 1:100 dilution; Novus Biologicals, Abingdon, UK) and Granzyme B (EPR8260, 1:200 dilution; Abcam) were prepared as per the manufacturer’s instructions.

### An immunohistochemical evaluation

**Fig. 2:**
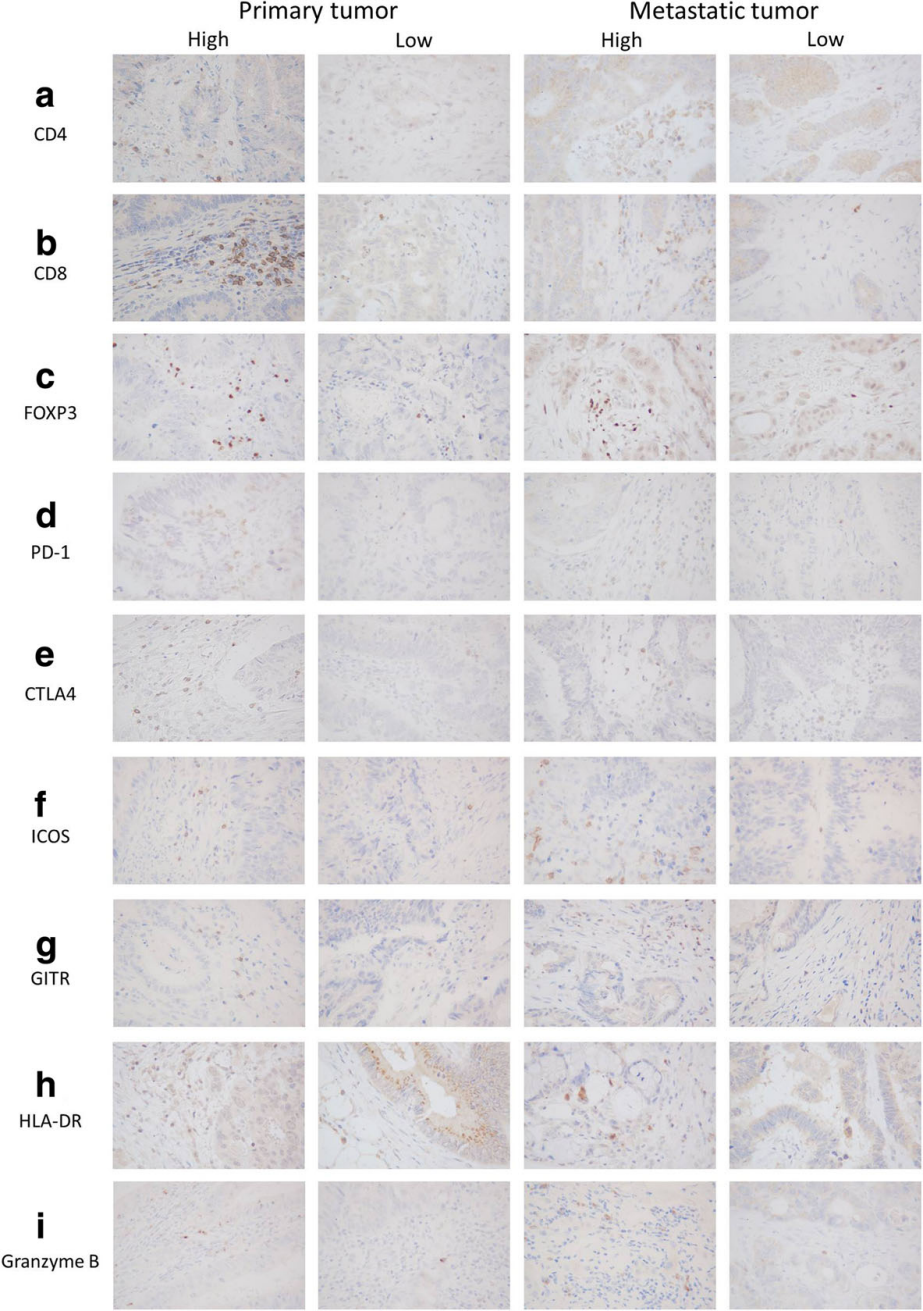
Representative pictures of each TIL subset by immunohistochemical staining (× 400). Tissue sections were immunostained with anti-CD4 (**a**), anti-CD8 (**b**), anti-FOXP3 (**c**), anti-PD-1 (**d**), anti-CTLA4 (**e**), anti-ICOS (**f**), anti-GITR (**g**), anti-HLA-DR (**h**) or anti-Granzyme B (**i**) monoclonal antibody

An immunohistochemical evaluation was carried out by two independent pathologists who were blinded to the clinical information. The number of immunoreactive lymphocytes at the invasive margin was counted with a light microscope in a randomly selected field at a magnification of 400 (Fig. 2). The mean of values obtained in five different areas was used for the data analysis. We then classified patients into high- and low-TIL subset groups according to each median value.

### Statistical analyses

The significance of the correlations between TILs and the clinicopathological characteristics were analyzed using Fisher’s exact test. Associations between the density of the TILs in the primary tumor and that in the metastatic tumor were evaluated by Fisher’s exact test, Wilcoxon’s test and Spearman’s rank correlation coefficient. All of the statistical analyses were conducted using the JMP® 13.0.0 software program (2016 SAS institute Inc., Cary, NC, USA). *P* values of < 0.05 were considered to indicate statistical significance.

## Results

### Patient characteristics

The characteristics of the patients who underwent concurrent resection of the primary tumor and liver metastases are listed in Table 1. The distribution of the number of liver metastasis was as follows: 1 in 16 patients, 2 in 4 patients, and ≥ 3 in 4 patients. The median size of the largest metastatic tumor was 2.4 cm (range: 0.4–8.0 cm).

**Table 1:** Characteristics of the patients who underwent concurrent resection of the primary tumor and liver metastases

Age (years)	
Median (range)	67 (33–78)
Gender	
Male	13
Female	11
Location of the primary tumor	
Colon	15
Rectum	9
Histological type	
Well/Moderately differentiated tubular adenocarcinoma	23
Mucinous	1
Number of liver metastases	
1	16
2	4
≥ 3	4
Maximum size of the metastatic tumor (cm)	
Median (range)	2.4 (0.4–8.0)

### Correlations between the density of TILs and the characteristics of the metastatic liver tumor

The densities of the TILs did not correlate with the characteristics of the metastatic liver tumor, such as the number and diameter (Table 2).

**Table 2:** Correlations between the density of TILs and the status of the metastatic liver tumor

	Number of metastatic tumors			Diameter of the metastatic tumor		
	1	> 1	*p*-value	< 2.4 cm	≥2.4 cm	*p*-value
Primary tumor inflammatory cells evaluated by HE-stained sections						
Low	10	5		8	7	
High	6	3	1.000	4	5	1.000
Primary tumor CD4^+^ TILs						
Low	8	4		6	6	
High	8	4	1.000	6	6	1.000
Primary tumor CD8^+^ TILs						
Low	8	8		7	5	
High	4	4	1.000	5	7	0.684
Primary tumor FOXP3^+^ TILs						
Low	8	4		5	7	
High	8	4	1.000	7	5	0.684
Metastatic tumor inflammatory cells evaluated by H-E stained sections						
Low	11	4		8	7	
High	5	4	0.412	4	5	1.000
Metastatic tumor CD4^+^ TILs						
Low	8	4		6	6	
High	8	4	1.000	6	6	1.000
Metastatic tumor CD8^+^ TILs						
Low	8	4		5	7	
High	8	4	1.000	7	5	0.684
Metastatic tumor FOXP3^+^ TILs						
Low	9	3		6	6	
High	7	5	0.667	6	6	1.000

*HE* Hematoxylin and eosin, *TILs* tumor-infiltrating lymphocytes, *FOXP3* Forkhead boxprotein P3

### Correlation between the density of TILs in the primary and metastatic tumors

**Fig. 3:**
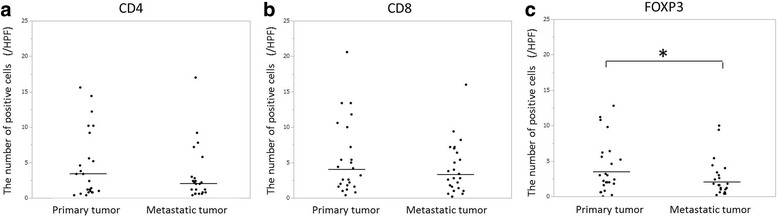
A comparison of the density of each TIL subset between the primary tumor and the metastatic tumor (**a**: CD4, **b**: CD8, **c**: FOXP3). For FOXP3 only, the density of TILs in the primary tumor was significantly higher than that in the metastatic tumor (*: *p* < 0.05)

**Fig. 4:**
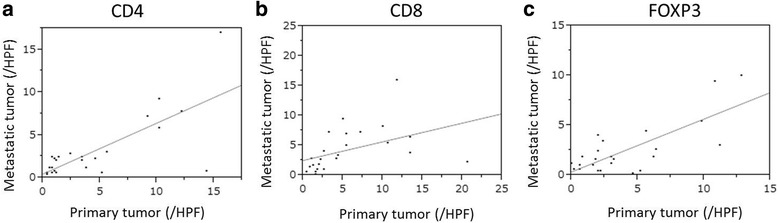
The correlation between the number of TIL subsets in the primary tumor and that in the metastatic tumor (**a**: CD4, **b**: CD8, **c**: FOXP3). The numbers of CD4^+^, CD8^+^ and FOXP3^+^ TILs in the primary tumor was significantly associated with those in the metastatic tumor (*r* = 0.634, *p* = 0.001, *r* = 0.700, *p* < 0.001, *r* = 0.559, *p* = 0.006, respectively)

According to the evaluation of TILs using the HE-stained sections, among the nine cases with high-TILs in the primary tumor, seven cases (77.8%) were judged as having high-TILs in the metastatic tumor. Furthermore, among the fifteen cases with low-TILs in the primary tumor, thirteen cases (86.7%) were judged to have low-TILs in the metastatic tumor. The density of TILs in the primary tumor was significantly associated with that in the metastatic tumor (*p* = 0.003) (Table 3). The median number of CD4^+^, CD8^+^, FOXP3^+^ TILs in the primary and metastatic tumors is shown in Table 4. Although the median value of each of the TIL subsets was higher in the primary tumor than in the metastatic tumor, significant differences were observed in only FOXP3 (Fig. 3). Furthermore, the number of CD4^+^, CD8^+^ and FOXP3^+^ TILs in the primary tumor was also significantly associated with these numbers in the metastatic tumor (*r* = 0.634, *p* = 0.001; *r* = 0.700, *p* < 0.001; *r* = 0.559, *p* = 0.006, respectively) (Fig. 4).

**Table 3:** Correlation between the density of TILs in the primary tumor and that of TILs in the metastatic tumor evaluated by HE-stained sections

	The density of TILs in the metastatic tumor		
	Low	High	*p*-value
The density of TILs in the primary tumor			
Low	13	2	
High	2	7	0.003

*TILs* tumor-infiltrating lymphocytes, *HE* hematoxylin and eosin

**Table 4:** The number of TIL subsets at the primary tumor and the metastatic tumor

	Primary tumor	Metastatic tumor
CD4		
Median (range)	2.9 (0.4–15.6)	2.1 (0.4–17.0)
CD8		
Median (range)	3.7 (0.4–20.6)	3.1 (0.2–16.0)
FOXP3		
Median (range)	2.7 (0–12.8)	1.7 (0.2–10.0)

*FOXP3* Forkhead boxprotein P3

### The correlation between the activation/suppression marker values in the primary and metastatic tumors

**Fig. 5:**
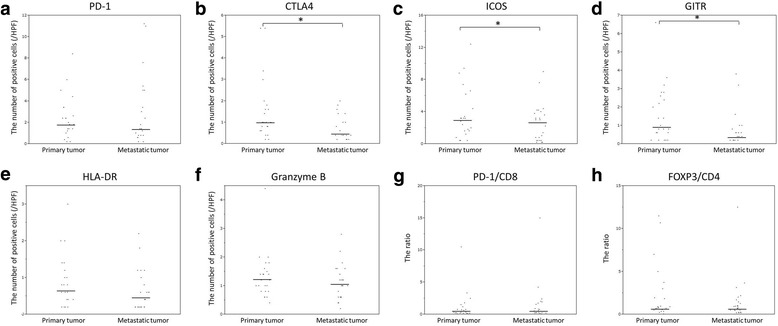
The comparison of the activation/suppression values of the primary and metastatic tumors (**a**: PD-1, **b**: CTLA4, **c**: ICOS, **d**: GITR, **e**: HLA-DR, **f**: Granzyme B, **g**: the PD-1/CD8 ratio, **h**: the FOXP3/CD4 ratio). The values of CTLA4, ICOS and GITR (activation/suppression markers) in the primary tumor were significantly higher than those in the metastatic tumor (*: *p* < 0.05)

**Fig. 6:**
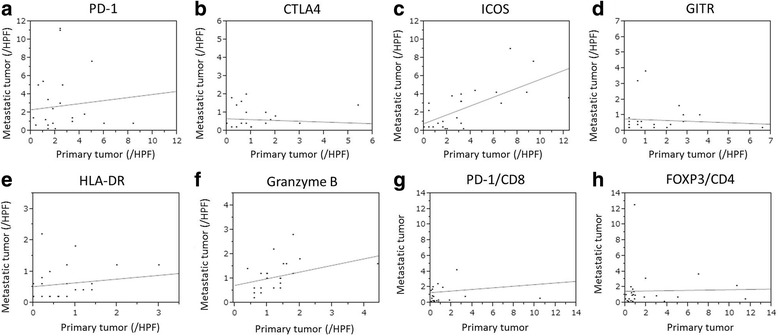
The correlation between the activation/suppression marker values of the primary and metastatic tumors (**a**: PD-1, **b**: CTLA4, **c**: ICOS, **d**: GITR, **e**: HLA-DR, **f**: Granzyme B, **g**: the PD-1/CD8 ratio, **h**: the FOXP3/CD4 ratio). The values of ICOS^+^, Granzyme B^+^ TILs, and the PD-1/CD8 ratio in the primary tumor were significantly correlated with those in the metastatic tumor (*r* = 0.649, *p* = 0.001, *r* = 0.426, *p* = 0.038, *r* = 0.498, *p* = 0.013, respectively)

The median activation/suppression marker values (including PD-1^+^, CTLA4^+^, ICOS^+^, GITR^+^, HLA-DR^+^, and Granzyme B^+^ TILs) in the primary and metastatic tumors are shown in Table 5. The median values of CTLA4^+^, ICOS^+^, and GITR^+^ TILs in the primary tumor were significantly higher than those in the metastatic tumor (Fig. 5). Furthermore, the number of ICOS^+^, Granzyme B^+^ TILs and the PD-1/CD8 ratio in the primary tumor were significantly correlated with the values in the metastatic tumor (*r* = 0.649, p = 0.001; *r* = 0.426, *p* = 0.038; *r* = 0.498, *p* = 0.013, respectively), and the number of PD-1^+^ TILs tended to be correlated with that in the metastatic tumor (*r* = 0.387, *p* = 0.062) (Fig. 6).

**Table 5:** The activation/suppression status of the lymphocytes of the primary tumor and the metastatic tumor

	Primary tumor	Metastatic tumor
PD-1		
Median (range)	1.8 (0–8.4)	1.3 (0–11.2)
CTLA4		
Median (range)	1.0 (0–5.4)	0.4 (0–2.0)
ICOS		
Median (range)	3.0 (0–12.4)	2.6 (0.2–9.0)
GITR		
Median (range)	0.9 (0–6.6)	0.4 (0–3.8)
HLA-DR		
Median (range)	0.6 (0–3.0)	0.4 (0–2.2)
Granzyme B		
Median (range)	1.2 (0.4–4.4)	1.0 (0–2.8)
The PD-1/CD8 ratio		
Median (range)	0.32 (0–10.5)	0.41 (0–15.0)
The FOXP3/CD4 ratio		
Median (range)	0.79 (0–11.5)	0.86 (0.2–12.5)

*PD-1* Programmed cell death 1, *CTLA4* cytotoxic T-lymphocyte-associated protein 4, *ICOS* inducible T-cell co-stimulator, *GITR* Glucocorticoid induced tumor necrosis factor receptor related protein, *HLA-DR* Human Leukocyte Antigen - antigen D Related, *FOXP3* Forkhead boxprotein P3

## Discussion

In this study, the local immune status of the primary tumor was found to be similar to that of the metastatic tumor in relation to the anticancer immunity. According to the evaluation using the HE-stained sections, the density of tumor-infiltrating mononuclear inflammatory cells in the primary tumor was significantly associated with that in the metastatic tumor. Furthermore, according to an evaluation using immunohistochemistry, the density of TIL subsets, such as CD4^+^, CD8^+^ and FOXP3^+^ TILs, in the primary tumor was significantly associated with that in the metastatic tumor. Furthermore, the density of PD-1^+^, ICOS^+^ and Granzyme B^+^ TILs, a marker of immune escape/activation, in the primary tumor was associated with that in the metastatic tumor.

TILs, which reflect the immune response of the host, are a useful biomarker for predicting the survival and therapeutic outcomes in patients with various cancers, including colorectal, breast, lung, gastric and esophageal cancer, and a number of reports on TILs have been published [11–17]. However, most of them concerned the TILs in the primary tumor, and very few reports have examined TILs in the metastatic tumor. As to the reason for this discrepancy in focus, resection of the metastatic tumor is included in the therapeutic strategy in CRC [18], while resection of the metastatic tumor is not included in the therapeutic strategy in various other types of cancer, such as gastric cancer and pancreatic cancer [19, 20]. Furthermore, the rate of surgical indication is quite low, even in cases of metastatic CRC [21]. Therefore, relatively few samples of metastatic tumor have been made available, resulting in markedly fewer reports on the TILs in metastatic tumors than on the TILs in primary tumors.

Control of the metastatic lesion is very important as a factor related to the prognosis in metastatic CRC. The treatment most often used for unresectable distant metastatic tumor is chemotherapy, and the density of TILs was reported to affect the chemotherapeutic effectiveness. Therefore, it is important to grasp the local immune status of the metastatic lesion in patients with unresectable metastatic CRC. However, it is not easy to collect tissue samples of the metastatic lesion with minimal invasion in clinical practice, making it difficult to grasp the local immune status of the metastatic lesion. In this study, the density of TILs in the metastatic tumor was revealed to have no relationships with the number or size of the metastatic tumor but was shown to be similar to the density of TILs in the primary tumor, as in a few past reports [5]. Furthermore, the activation/suppression status of the lymphocytes in the primary tumor was similar to that in the metastatic tumor. Although it did not apply to all of the markers, some of the activation/suppression marker values (i.e., ICOS and Granzyme B) of the primary tumor were significantly correlated with those of the metastatic tumor. In addition, the PD-1/CD8 ratio—which has been reported to reflect the immunosuppressive status in the cancer microenvironment [10] in the primary tumor was correlated with that in the metastatic tumor. Thus, it was considered that in addition to the density of the TIL subsets, the activation/suppression status of the lymphocytes of the primary tumor was associated with that of the metastatic tumor. These results supported our claim that the local immune statuses of the primary and metastatic tumors were associated with each other. Thus, the density of TILs in the primary tumor may be a surrogate marker for the density of TILs in the metastatic tumor. The results obtained in this study support the claim that the local immune status of the primary tumor is associated with the efficacy of the chemotherapy, which was reported in our previous report [22]. It may be possible to predict the chemotherapeutic efficacy on the metastatic tumor by evaluating the density of TILs in the primary tumor, even if the local immune status of the metastatic tumor cannot be evaluated. The evaluation of the density of TILs in the primary tumor may enable clinicians to stratify the patients who can expect the conversion surgery or the patients who need intensive chemotherapy.

In previous reports, the number of the TILs in the primary tumor was reported to be greater than that in the metastatic tumor. In the present study, the median number of TIL subsets in the primary tumor was greater than that in the metastatic tumor. However, a significant difference was found with respect to FOXP3. We considered that this was because of the small number of cases. On the other hand, the comparison of the activation status of the lymphocytes between the primary tumor and the metastatic tumor was not clarified in this study. Both of the activation and the suppression marker values in the primary tumor were greater than those in the metastatic tumor. This is a subject for future analysis.

There are several points to consider in evaluating the density of TILs in the metastatic tumor in this study. First, the patients with disease recurrence after the curative resection of the primary tumor or the patients who underwent two-stage hepatectomy for synchronous liver metastasis were excluded in this study, because the cancer microenvironment, including the local immune status may change with the lapse of time [23]. That is, in this study, only the patients who underwent concurrent resection of the primary tumor and the metastatic lesion were enrolled. Second, only the patients with no neoadjuvant therapy were enrolled in this study, because the local immune status may change in response to chemotherapy and radiotherapy [24, 25]. Third, the same organ should be chosen when evaluating the density of TILs in the metastatic tumor, as the density of TILs may differ depending on the metastatic organ [26]. Therefore, the subjects were limited to the patients with liver metastases in this study.

Several limitations associated with the present study warrant mention. First, we evaluated a relatively small number of patients, and the study design was retrospective. Second, as mentioned in the previous reports regarding TILs, problems such as the heterogeneity and the optimal cut-off value remain unsolved. Third, whether or not all metastatic tumors have a similar immune status remains unclear, as we evaluated only the largest tumor in cases of multiple liver metastases.

## Conclusions

The local immune status of the primary tumor was revealed to be similar to that of the metastatic tumor. This suggests that the evaluation of the local immunity of the primary tumor may be a substitute for the evaluation of the local immunity of the metastatic lesion. Therefore, information on the primary tumor may be useful when considering treatment strategies for metastatic lesions.

## Abbreviations

CRC: Colorectal cancer
CTLA4: Cytotoxic T-lymphocyte-associated protein 4
FOXP3: Forkhead boxprotein P3
GITR: Glucocorticoid-induced tumor necrosis factor receptor related protein
HE: Hematoxylin and eosin
HLA-DR: Human Leukocyte Antigen - antigen D Related
ICOS: Inducible T-cell co-stimulator
PD-1: Programmed cell death 1
TILs: Tumor-infiltrating lymphocytes
